# Supplementary material for: Brain Glucose Metabolism and COMT Val 158 Met Polymorphism in Female Patients with Work-Related Stress
Source: Diagnostics (Basel). 2024 Aug 9;14(16):1730. doi: 10.3390/diagnostics14161730 (PMC11353128; doi:10.3390/diagnostics14161730)
Supplement: Supplementary file 1 [file diagnostics-14-01730-s001.zip › Supporting Information S1_ Screening for stress symptoms.pdf]

Supplementary Materials S1: Screening for stress symptoms

**Stress symptoms**	
Somatic and physical symptoms	Inner turmoil<br>Headaches<br>Tingling in arms<br>Palpitations<br>Upset stomach<br>Abdominal pains<br>Decreased potency<br>Weight loss or weight gain<br>Frequent infections<br>Aggravation of other chronic conditions<br>Dizziness<br>Visual and auditory disturbances
Mental symptoms	Anhedonia<br>Fatigue<br>Irritability<br>Memory problems<br>Difficulty concentrating<br>Anxiety<br>Depressed mood<br>Worrying<br>Confusion
Behavioral symptoms	Insomnia<br>Apathy<br>Hyperventilation<br>Aggressiveness<br>Reduced performance<br>Indecisiveness<br>Feeling of powerless-ness<br>Self-medication
