# Supplementary material for: Brain Glucose Metabolism and COMT Val 158 Met Polymorphism in Female Patients with Work-Related Stress
Source: Diagnostics (Basel). 2024 Aug 9;14(16):1730. doi: 10.3390/diagnostics14161730 (PMC11353128; doi:10.3390/diagnostics14161730)
Supplement: Supplementary file 1 [file diagnostics-14-01730-s001.zip › Supporting Information S2_ Summary of SCAN interview.pdf]

Supplementary Materials S2: Summary of Schedules for Clinical Assessment in Neuropsychiatry (SCAN)

The SCAN interview is used to assess the present state and current conditions of the project participants.

Please fill out the questionnaire. When filling out the form, think about the time of the past week.

Rating Scale

0: The symptom is not present

1: The symptom is present in a mild to moderate degree

2: The symptom is present in a severe or pronounced degree

?: Not sure if the symptom is present after relevant questioning

-: Cannot be rated (can or will not answer, not asked)

Date of sick leave

Physical Health

Somatic disease or dysfunction

1. Do you have any physical illness or disorder, pains or weaknesses?

Gastrointestinal symptoms

Abdominal pains

Nausea

Vomiting

Feeling of bloating

Frequent or loose stools, blood, or mucus from the rectum

Coated tongue or bad taste in the mouth

Cardiovascular symptoms

2. Shortness of breath

Urogenital symptoms

3. Dysuria (pain / burning) or difficulty urinating

4. Genital pain or discomfort
5. Abundant or unusual discharge

Skin and pain symptoms

6. Skin spots or discoloration
7. Headache, back pain, extremities or joints

Neurological symptoms

8. Dizziness, fainting or cramps
9. Steering or balance difficulties
10. Paralysis, loss of strength, aphonia (toneless voice)
11. Double vision, blindness, deafness
12. Decreased feeling or pain, feeling of death, tingling

Autonomic symptoms

13. Excessive heartbeat
14. Sweden
15. Dry mouth
16. Flush or flare
17. Butterflies

Preoccupation with symptoms

18. Are the symptoms bothersome?
19. Have you sought medical attention or had examinations performed?
20. How often?

Lack of confidence in the doctor's reassuring statements

21. What do you think about what the doctor said?
22. Were you satisfied?

Hypochondriac preoccupation

23. Do you think you have a serious physical illness or deformity?

Which one?___________________________________

Excessive physical fatigue

24. Do you feel exhausted after a minimum of physical exertion?
25. Does it help to rest or relax?

Physical disability

26. Has it affected your ability to work or your social life?
27. Has it affected your ability to cope with practical and personal necessities in daily life?

Dissociative symptoms

Problems with memory, identity or mental control over the body. There are also a large number of other emotional and mental problems that may arise in this regard.

Cases of amnesia

28. Have you had cases of complete or partial memory loss?

Fugue

29. Have there been situations where you have moved or traveled around without remembering anything about it?

Trance or obsession experiences?

30. Have you experienced being in a trance?
31. Have you been possessed by a spirit or power?

Eating disorders

Knowledge of different types of eating disorders, their symptoms, causes and other concomitant disorders.

Weight in kg

____

Height in cm

____

Fear of being or getting too fat

32. Do you feel overweight or obese?

33. Are you afraid of getting fat?

Eating cravings

34. Fear of being or getting too fat

Do you avoid fatty foods?

35. Do you follow a special diet?

Activities to lose weight

36. Do you exercise a lot?
37. Are you trying to get rid of food by throwing up?
38. Do you use laxatives, weight loss pills or other medications?

Sexual dysfunction

Decreased or lack of sexual desire or the like.

Sexual dysfunction

39. Do you lack the desire, pleasure or ability to have sex?
40. Is sexual intercourse uncomfortable or painful?

Tension and tendency to worry.

41. Have you been worrying more than usual?

Feeling of nervous tension

42. Do you feel nervous, tense?
43. Do you have your nerves up?

Generalized muscle tension

44. Do you find it difficult to relax?
45. Do your muscles feel tense?

Localizing tension pain

46. Do you have pain or soreness in the muscles?
47. Headache?

48. Neck or back pain?

Tendency startle

49. Have you become prone to tension when you are startled or surprised?

Anxiety:

Fear, tension or restlessness accompanied by autonomic symptoms

Feeling anxious

50. Have you felt scared or anxious?
51. Have you had any panic attacks?

Phobias

52. Does anxiety occur in special situations that you may be trying to avoid?

Autonomic symptoms:

53. Palpitations, restlessness or fast pulse?
54. Are you sweating?
55. The tremor or the tremor?
56. Feeling of dryness in the mouth?

Symptoms from chest and abdomen

57. Difficulty breathing?
58. Feeling of suffocation?
59. Pain or restlessness in the chest?
60. Nausea or upset stomach?

Mental symptoms

61. Uncertainty feeling or fainting?
62. Feeling unreal?
63. Fear of losing self-control?
64. Fear of death?

General symptoms

65. Chills or hot flashes?

66. Unable to feel or tingling in the skin?
67. Difficulty swallowing or lump in the throat?

Panic attacks with autonomic symptoms

68. Have you had sudden bouts of anxiety or panic where you felt you simply had to do something to make it stop?

Free anxiety with autonomic symptoms.

69. Have you had anxious feelings for no reason for longer periods?

Anxious beforehand with autonomic symptoms.

70. Have you felt anxious about, that something terrible could happen to yourself or your loved ones?

Agoraphobic anxiety.

Do you experience panic anxiety when you find yourself in situations where it is difficult to get away or get help, e.g.:

71. In crowds?
72. In public places?
73. On the move alone?
74. When traveling outside the home?

Monophobia anxiety.

Do you become anxious in other special situations, e.g. in connection with:

75. Animals, insects?
76. Altitudes, flight?
77. Thunderstorm, darkness?
78. Small and enclosed spaces?
79. The sight of blood?
80. Dental visits?

Avoidance behavior

81. Are you looking to avoid these situations or just avoiding thinking about them?

Obsessive symptoms

Obsessive thoughts and actions

Obsessive thoughts

82. Have you had unpleasant thoughts or impulses that come back even though you are trying to keep them out?
83. Can't you free yourself from having to think about certain topics all the time?

Coercive acts (control and repetition compulsion).

84. Do you need to check e.g. if you have locked or turned off even though you know you have already done so?
85. Do you have to repeat certain actions repeatedly?
86. To count or touch certain things?

Coercive acts associated with order

87. Do you have to keep everything in a certain order?
88. Do you have to follow certain rituals?

Coercive measures associated with cleanliness.

89. Do you have to wash your hands or take a bath repeatedly, perhaps for fear of infection or dirt?

Subjective resistance

90. Have you tried to resist or reject the thoughts or actions?
91. What happens then?

____

(Explain briefly, what is happening.)

Subjective recognition of unreasonableness.

Depressive symptoms

Immersion of the mood bed the pervasive and persistent emotional state of mind.

Depression

92. Have you felt sad or depressed?

Cheerlessness, reluctance

93. Have you had a hard time rejoicing over something?
94. Have you lost the desire to do anything?

Loss of interest

95. Have you lost interest in what you are usually interested in?

Reduced energy

96. Have you lost interest and energy?

Crying tendency

97. Do you cry easily?

Feeling of hopelessness

98. How do you feel about your future?
99. Can you see bright spots or hope ahead?
100. Does it all seem hopeless?

Preoccupation with thoughts of death and misfortune

101. Do you think a lot about possible accidents and death, ruin or destruction?

Thoughts of death

102. Have you felt that life was not worth living?
103. Did you wish you were dead?

Suicidal thoughts

104. Have you had thoughts of committing suicide?
105. Have you planned or tried to do that?
106. Do your feel less self-confident?

Feeling of numbness

107. Have you felt incompetent or inferior?
108. That you do meet expectations or are completely incompetent?

Social withdrawal

109. Have you become more withdrawn, inclined to keep to yourself?

110. Do not pick up the phone when it rings?

111. Do you not open when someone comes?

Universal pessimism

112. Universal pessimism. Does it all look sad and impossible in the future, no matter what you think?

113. Are you pondering the past?

Pathological guilt

114. Have you blamed yourself for something you have done?

115. Have you felt guilty or ashamed?

Guilty self-attribution.

116. Have you felt that others also blamed you or accused you of what you had done?

Increased mental fatigue and exhaustion.

117. Have you felt inexplicably tired?

118. Or are you getting tired fast?

119. Do you feel exhausted after the slightest mental effort?

Feeling of insufficiency.

120. Does it all feel overwhelming?

121. Heavy or unaffordable?

122. Do you have to pull yourself together if you have to do something?

Difficulty thinking

123. Do you find it difficult to think clearly and coherently?

124. Do you find it difficult to make decisions?

Difficulty concentrating

125. Do you have difficulty gathering your thoughts, e.g. read a book or watch a movie?

Subjective inhibition

126. Do you feel you have become slower in all you do?

Subjective taciturnity

127. Have you become more silent, speak less?

Restlessness

128. Do you feel uneasy inside or restless?
129. So restless that you have to walk up and down the floor all the time?

Appetite change

130. Have you lost your appetite?
131. Or have you had more appetite than usual?

Weight change

132. Have you lost or gained weight?

Decreased libido

133. Have you lost your desire for sex?

Sleep disorders

134. Do you have difficulties falling asleep?
135. Do you have difficulties staying asleep throughout the night?
136. Do you need to sleep more than you usually do?

Early awakening

137. Do you wake up several hours before you usually do and can no longer sleep?

Morning aggravation

138. When is the condition worst?

Non-reactive depression

139. Do you feel more or less bad all the time - even if you get a visit or are preoccupied with something?

Manic symptoms

140. Mania is a condition in which one is morbidly elated.

Expansive mood rental

141. Have you felt elated, overjoyed or excited?

Irritable mood

142. Do you constantly feel irritated towards others?
143. Tendency to be on edge all the time?

CUT OFF Mani

Perception disorders

A perception disorder is an abnormal interpretation of sensory impressions.

Disturbed perception

144. Has there been anything unusual about the way things look?

Derealization

145. Have you had a feeling that everything was all unreal?

Depersonalization

146. Have you felt unreal, like "not really" present?

CUT OFF Perception

Hallucinations

A hallucination is when one sees, hears, smells, tastes or feels something on the skin that is not there in reality.

Hearing hallucinations

147. Did you hear voices or sounds when there was no one or anything present that explained it?
148. Or that others could not hear?

Other hallucinations

149. Have you had visions that others could not see?

150. Or experienced odors, tastes, or physical sensations that were not explainable?

CUT OFF Hallucinations

Subjective thought disorders

Subjective thought disorders are a group of phenomena that for the most part are a combination of delusions and an experience that the mind is disturbed in an unnatural way.

Disturbance of thoughts

151. Can you think clearly?

152. Is there anything that is disturbing or affecting your thoughts?

Feeling we have 'Run out of gas' emotionally?

153. Can anyone read your thoughts?

How do they do it?________________________________

(Explain briefly how you think others can read your thoughts.)

CUT OFF Thought Disorders

Experience of promoted control

154. Have you experienced that your will was replaced by another will, so that you were ruled from without by an alien force or power?

CUT OFF Steering experience

Misconception

155. Have you had a feeling or sensation that something strange or inexplicable was going on around you?

Self-referential performances and pursuit performances

156. Have you been the subject of remarkable attention?

157. Did you receive the message through TV or something else?

158. Have you been persecuted in general?

CUT OFF Delusions

Use of alcohol and other psychoactive substances that is substances that affect the brain.

Alcohol use:
Average weekly consumption for men> 21 items, for women> 14 items are considered as high-risk consumption.

Abuse of prescribed medication:
Use of other or more medication than prescribed.

Substance abuse: Abuse of other drugs. When abusing several psychoactive substances, only one or is rated no more than two of the most commonly used or significant substances.

Abuse of alcohol, drugs or drugs.

159. Do you consume too much alcohol or medication?

Cognitive symptoms
Problems with thinking, lack of concentration and problems with memory.

Cognitive impairment

160. Have you had memory problems?
161. Problems with attention or perception?
162. Problems finding words, writing or counting?
163. Problems with finer movements e.g. buttons and laces?
