# Supplementary material for: Brain Glucose Metabolism and COMT Val 158 Met Polymorphism in Female Patients with Work-Related Stress
Source: Diagnostics (Basel). 2024 Aug 9;14(16):1730. doi: 10.3390/diagnostics14161730 (PMC11353128; doi:10.3390/diagnostics14161730)
Supplement: Supplementary file 1 [file diagnostics-14-01730-s001.zip › Supporting Information S4_ Cluster parameters.pdf]

Supplementary Materials S4: Mean values cluster

Cluster size	Peak t/t	x,y,z (mm)	Neuromorphometrics	Mean cluster estimates stress patients	Mean cluster estimates HC
Clusters identified in the one-tailed t-tests HC > stress patients					
6	4.15	-30 24 24	Middle frontal gyrus left	0.9072<br>0.7950<br>0.9542<br>0.8395<br>0.9836<br>0.9158<br>0.7672<br>0.5864<br>0.5868<br>0.8642	1.2757<br>1.6481<br>1.4083<br>1.1140<br>1.1822<br>1.0057<br>1.3679<br>1.4005
Total mean Max cluster				0.8200	1.3003
4	3.98	28 8 44	Middle frontal gyrus right	1.4470<br>0.9646<br>1.2222<br>1.2167<br>0.9228<br>1.0856<br>1.3152<br>0.6812<br>0.6422<br>1.1053	1.3759<br>2.2036<br>2.2646<br>1.7842<br>2.0298<br>2.0683<br>1.2778<br>1.7061
1	3.70	12 -16 68	Precentral gyrus right	0.9724<br>1.1523<br>1.4199<br>1.1439<br>1.1743<br>1.4043<br>1.2483<br>1.0828<br>1.1334<br>1.2625	1.3840<br>2.3938<br>2.1255<br>1.8086<br>1.6879<br>1.8177<br>1.8355<br>1.4904
